# Cytokines in atherosclerosis: Key players in all stages of disease and promising therapeutic targets

**DOI:** 10.1016/j.cytogfr.2015.04.003

**Published:** 2015-12

**Authors:** Dipak P. Ramji, Thomas S. Davies

**Affiliations:** Cardiff School of Biosciences, Cardiff University, Sir Martin Evans Building, Museum Avenue, Cardiff CF10 3AX, UK

**Keywords:** Atherosclerosis, Cytokines, Chemokines, Inflammation, Therapeutic avenues, ABC, ATP-binding cassette, ACAT-1, acyl-CoA acyl transferase-1, ADRP, adipocyte differentiation related protein, Apo, apolipoprotein, BMT, bone marrow transplantation, CANTOS, Canakinumab Anti-inflammatory Thrombosis Outcomes Study, CIRT, Cardiovascular Inflammation Reduction Trial, CCR2, CC-chemokine receptor-2, CR, chemokine receptor, CVD, cardiovascular disease, CSF, colony-stimulating factor, DCs, dendritic cells, ECs, endothelial cells, ECM, extracellular matrix, eNOS, endothelial nitric oxide synthase, ER, endoplasmic reticulum, ERK, extracellular signal-regulated kinase, Fn14, fibroblast growth factor-inducible 14, G-CSF, granulocyte colony-stimulating factor, GDF-15, growth differentiation factor-15, GM-CSF, granulocyte macrophage colony-stimulating factor, ICAM-1, intercellular adhesion molecule-1, IFN, interferon, IL, interleukin, IL-18BP, IL-18 binding protein, IL-1RA, IL-1 receptor antagonist, LDL, low-density lipoprotein, LDLr, low-density lipoprotein receptor, LFA1, lymphocyte function-associated antigen 1, LIGHT, homologous to lymphotoxin, exhibits inducible expression, and competes with HSV glycoprotein D for herpes virus entry mediator, a receptor expressed by T lymphocytes, M-CSF, macrophage-colony stimulating factor, MHC, major histocompatibility complex, MIF, macrophage migration inhibitory factor, miRNA, micro RNA, mmLDL, minimally modified LDL, MMP, matrix metalloproteinase, LXR, liver X receptors, NK, natural killer, NOD, nucleotide-binding oligomerization domain, NLR, NOD-like receptor, NLRP3, NLR family, pyrin domain containing 3, NPC, Niemann-Pick disease, type C, OxLDL, oxidized LDL, PAI, plasminogen activator inhibitor, PECAM-1, platelet endothelial cell adhesion molecule-1, PRR, pattern recognition receptor, RCT, reverse cholesterol transport, ROS, reactive oxygen species, SMCs, smooth muscle cells, SOCS, suppressor of cytokine signaling, SR, scavenger receptor, SREBP, sterol response element binding protein, SR-PSOX, SR that binds phosphatidyl serine and oxidized lipoprotein, STAT1, signal transducer and activator of transcription-1, TF, tissue factor, TGF, transforming growth factor, Th, T-helper, TIMP, tissue inhibitor of metalloproteinases, TNF, tumor necrosis factor, TL1A, TNF-like protein 1A, TNFSF12, TNF superfamily member 12, TLR, Toll-like receptor, TRAIL, TNF-related apoptosis-inducing ligand, Tregs, regulatory T cells, TWEAK, TNF-related weak inducer of apoptosis, VCAM-1, vascular cell adhesion molecule-1, VLA4, very late antigen 4, wt, wild type

## Abstract

Atherosclerosis, a chronic inflammatory disorder of the arteries, is responsible for most deaths in westernized societies with numbers increasing at a marked rate in developing countries. The disease is initiated by the activation of the endothelium by various risk factors leading to chemokine-mediated recruitment of immune cells. The uptake of modified lipoproteins by macrophages along with defective cholesterol efflux gives rise to foam cells associated with the fatty streak in the early phase of the disease. As the disease progresses, complex fibrotic plaques are produced as a result of lysis of foam cells, migration and proliferation of vascular smooth muscle cells and continued inflammatory response. Such plaques are stabilized by the extracellular matrix produced by smooth muscle cells and destabilized by matrix metalloproteinase from macrophages. Rupture of unstable plaques and subsequent thrombosis leads to clinical complications such as myocardial infarction. Cytokines are involved in all stages of atherosclerosis and have a profound influence on the pathogenesis of this disease. This review will describe our current understanding of the roles of different cytokines in atherosclerosis together with therapeutic approaches aimed at manipulating their actions.

## 1 Introduction

Cardiovascular disease (CVD) is responsible for most mortality worldwide and accounted for about 31.9% of all deaths in 2010 in the United States alone [1]. The total direct and indirect costs from CVD in 2010 were estimated at $315.4 billion [1]. Although morbidity and mortality from CVD has decreased in the last two decades, at least in the western world, it is expected that this will reverse in the future because of a global increase in diabetes and obesity along with an alarming rise in CVD in developing countries in part due to acquisition of a westernized lifestyle.

**Fig. 1:**
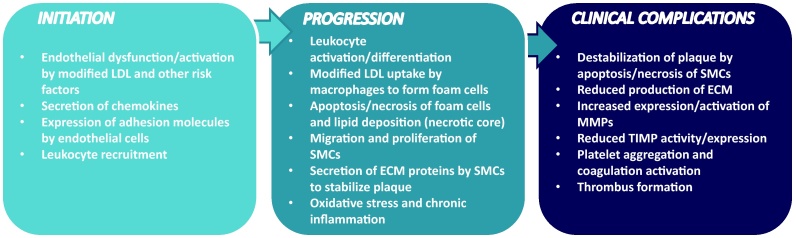
*Pathogenesis of atherosclerosis.* The disease is initiated by the activation of the endothelium/endothelial cell (EC) dysfunction by accumulation of LDL, which subsequently gets modified (e.g. oxidized), together with other atherogenic factors. The activated ECs secrete a range of chemokines and increase the expression of adhesion proteins on their cell surface. This results in the recruitment and infiltration of immune cells such as monocytes. The monocytes differentiate into macrophages, which is accompanied by increased expression of pattern recognition receptors on their surface, which participate in the promotion of inflammation and uptake of modified LDL, leading to the formation of lipid laden foam cells. Continued accumulation of modified LDL together with disturbed cellular lipid homeostasis causes apoptosis/necrosis of foam cells resulting in lipid deposition (necrotic core) and amplification of the inflammatory response. Smooth muscle cells (SMCs) migrate from the media to the intima where they proliferate, uptake modified lipoproteins and secrete extracellular matrix (ECM) proteins that stabilizes the plaques (fibrous cap). Continued inflammation orchestrated by cytokines destabilizes such plaques via decreased production of ECM proteins (reduced synthesis together with apoptosis/necrosis of SMCs/SMC-derived foam cells), increased production/activities of ECM degrading matrix metalloproteinases (MMPs) and reduced expression/activities of inhibitors of these enzymes. Plaque rupture leads to platelet aggregation, coagulation and thrombus formation that ultimately results in the clinical complications associated with this disease. Cytokines affect all the different stages in the pathogenesis of atherosclerosis (see text for details). Abbreviations: ECM, extracellular matrix; LDL, low-density lipoprotein; MMP, matrix metalloproteinase; SMC, Smooth muscle cells; TIMP, tissue inhibitor of metalloproteinase.

Atherosclerosis, the underlying cause of myocardial infarction, cerebrovascular accident or peripheral vascular disease, is the major cause of deaths from CVD. Atherosclerosis is now recognized as an inflammatory disorder of medium and large arteries initiated by risk factors such as high plasma cholesterol levels and hypertension [2]. Atherosclerosis develops during the lifespan of an individual and involves a number of steps: activation of the endothelium and recruitment of immune cells; monocyte differentiation and foam cell formation; development of fibrotic plaques due to death of foam cells and migration and proliferation of smooth muscle cells (SMCs); and plaque rupture and thrombosis [2] (Fig. 1). Both the innate and adaptive immune response in atherosclerosis is orchestrated by a range of cytokines, which regulate all stages of the disease [2], [3], [4].

Cytokines are a diverse group of low-molecular weight proteins with over 100 identified so far. Cytokines are clustered into several classes such as the interleukins (IL), chemokines, colony-stimulating factors (CSF), tumor necrosis factors (TNF), the interferons (IFN) and transforming growth factors (TGF) [2], [3], [4]. Many cytokines are expressed in atherosclerotic plaques and all cells involved in the disease are capable of producing cytokines and responding to them [2]. They can be generally classified as pro- or anti-atherogenic though the roles of some are not as clear-cut and often context-dependent [2], [3], [4]. This review will discuss the roles of key cytokines in different stages of atherosclerosis. Although *in vitro* studies using cell culture model systems have made a major contribution in advancing our understanding of the roles of cytokines in the cellular processes associated with atherosclerosis, the major focus of this review will be on the outcome from studies using animal model systems. In particular, two mouse models, the apolipoprotein (Apo) E-deficient mice and the low-density lipoprotein receptor (LDLr)-deficient mice [5], [6], have been particularly useful in advancing our understanding of the molecular basis of atherosclerosis and the roles of various cytokines in the disease [2], [3], [4]. The use of bone marrow transplantation (BMT) approaches in such models also informs on whether a particular phenotype is driven by hematopoietic or non-hematopoietic cells [5], [6]. These mice can spontaneously form atherosclerotic lesions on a standard chow diet but feeding of a high fat, western-type diet can markedly speed up the development of the disease [5], [6]. It should be noted that caution needs to be exerted in the extrapolation of outcomes from such mouse models to humans because of many differences between the two species, including in lipoprotein metabolism and the inflammatory response [7], [8]. In addition, existing mouse models are not particularly useful for investigating the steps involved in the clinical complications of the disease, plaque rupture [7]. It is therefore important that wherever possible key findings are analyzed in the human context.

## 2 Initial stages of atherosclerosis: key roles for chemokines

Atherosclerotic plaques tend to form particularly at the inner curvatures and branch points of arteries that are often associated with disturbed blood flow, and is augmented by other factors such as high plasma low-density lipoprotein (LDL) concentration, hypertension and toxins from cigarette smoke [2]. The mechanical forces associated with blood flow have a profound effect on the properties of endothelial cells (ECs) of the arteries [9]. Thus, shear stress generally triggers an anti-atherogenic gene expression and signal transduction profile that is lost at sites of disturbed blood flow [9]. In addition, sites with disturbed blood flow are associated with changes in the morphology of ECs, increase in the permeability to macromolecules such as LDL, and accumulation of extracellular matrix (ECM) that causes the retention of such particles [9]. Cytokines can modulate EC permeability [2], [3]. For instance, IFN-γ and TNF-α cause reorganization of the actin and tubulin cytoskeletons in ECs, thereby opening up gaps between adjacent cells [10].

The activated ECs release a range of chemokines and other cytokines that then cause the recruitment of circulating immune cells, particularly monocytes and T lymphocytes [2]. In addition, the ECs express adhesion proteins, such as intercellular adhesion molecule-1 (ICAM-1) and vascular cell adhesion molecule-1 (VCAM-1), that participate in the recruitment of immune cells [2].

Chemokines are a large family of structurally related, chemoattracting cytokines that are divided into subgroups on the basis of the position of the amino terminal cysteine residues (CC, CXC, CX_3_C, XC) [11], [12], [13]. Chemokines interact with receptors that activate heterotrimeric G proteins and associated intracellular signaling pathways [11], [12]. The roles of chemokines in atherosclerosis, particularly in the recruitment of monocytes, has been reviewed extensively [14], [15], [16], [17] and hence is only briefly addressed here. Monocytes in the blood are generally classified in mice as the most abundant classical Lys6C^high^ [express high levels of CC-chemokine receptor (CCR)-2, believed to be pro-inflammatory in nature and their levels increase in hyperlipidemia) and less frequent Ly6C^low^ [express CX_3_C-chemokine receptor 1 and perceived to carry out a homeostatic function) [14], [17]. The recruitment of monocytes occurs in a number of stages: capture and rolling; arrest; and extravasation [14], [15], [16]. The capture and rolling phase for Lys6C^high^ monocytes involves immobilization of CCL5 and CXCL1 on proteoglycans and P-selectins on ECs [14], [15], [16]. These chemokines interact with receptors expressed on the surface of invading monocytes [14], [15], [16]. The firm adhesion of monocytes to ECs requires binding of adhesion molecules on ECs to integrins on monocytes: VCAM-1 to integrin α4β1 (very late antigen 4 or VLA4) and ICAM-1 to αLβ2 (lymphocyte function-associated antigen 1 or LFA1) [14], [15], [16]. The transmigration of monocytes across the endothelium is mediated by chemokines produced by not only ECs but other cells present in the lesion such as SMCs and emigrated leukocytes along with VCAM-1 and platelet endothelial cell adhesion molecule-1 (PECAM-1) [14], [15], [16].

The roles of various chemokines and their receptors in atherosclerosis have been analyzed in mouse model systems, and the outcomes are summarized in Table 1, Table 2. It should be noted that different studies have not always produced a consistent outcome (e.g. CCR7) (Table 1, Table 2). In addition, there is often a marked functional redundancy in the action of various chemokines with further complexity produced by the ability of some of them to interact with multiple receptors together with individual receptors having many ligands [11], [12], [13]. In addition, there are likely to be temporal expression pattern and function of some chemokines during different stages of atherogenesis [11], [12], [13]. Overall, the three most prominent chemokine:chemokine receptors involved in the transmigration of Ly6C^high^ monocytes include CCL2:CCR2, CX_3_CL1:CX_3_CR1, and CCL5-CCR5 [14], [73]. Deficiency of these three-chemokine action leads to almost complete attenuation of atherosclerosis in mouse model systems [74]. The entry of patrolling Ly6C^low^ monocytes occurs less frequently and tends to rely on the CX_3_CL1:CX_3_CR1 axis [14].

Although chemokines were originally discovered in relation to their roles in directing leukocytes to sites of inflammation, they are now known to play a number of other important functions in the disease [11], [12], [13]. For example, CX_3_CL1 can also serve as an adhesion molecule [33]. CCL2 also impairs reverse cholesterol transport (RCT) by repressing the expression of key proteins implicated in the efflux of cholesterol from cells [75]. Manipulating the chemokine:chemokine receptor axis represents a promising therapeutic avenue [11], [12], [13]. Indeed, existing therapies such as statins have been found to attenuate the expression of chemokine and their receptors [76]. Other approaches that have shown promise, at least in mouse model systems, are: manipulating sialylation that affects the interaction of chemokines with their cognate receptors [77]; antagonists/inhibitors of specific receptors [49], [65], [71]; nano-particle-facilitated gene silencing [50]; broad spectrum inhibitors such as viral proteins [78], [79], [80]; blocking antibodies against chemokines or their receptors [11], [12], [13]; decoy ligands that bind to the receptor but does not cause activation (e.g. met-RANTES) [11], [12], [13]; and molecules that target chemokine oligomerization [11], [12], [13]. Targeting multiple chemokines will be necessary given the independent roles of some chemokines [62], [81]. It is also important to point out that not all chemokines are pro-atherogenic. For example, CXCL5 limits macrophage foam cell formation in mouse model systems [27].

Deficiency of many cytokines (e.g. IL-1RA, IL-10, IL-13, IL-18, IL-19) is also associated with reduced macrophage recruitment (see Table 3, Table 4 for a summary of the outcome of studies on the roles interleukins and other cytokines respectively in mouse model systems). Induced expression of chemokines and adhesion molecules by ECs represents a major mechanism for such an action of these cytokines [2], [3].

## 3 Foam cell and fatty streak formation: Key roles for cytokines

The normal function of monocyte recruitment in inflammation is to participate in its resolution in order to ultimately decrease further entry of immune cells, cause egress of existing cells from inflammatory sites and efferocytosis of apoptotic cells [2], [14]. However, these homeostatic mechanisms are impaired in atherosclerosis resulting in a continuous recruitment of monocytes and reduced egress and efferocytosis of existing cells [2], [14]. In addition, local proliferation dominates macrophage accumulation in atherosclerotic plaques [197].

In the arterial intima, monocytes can differentiate to macrophages or dendritic cells (DCs) depending on the cytokine signal received with macrophage colony-stimulating factor (M-CSF) a major facilitator of macrophage differentiation [2]. Recent studies are highlighting the polarization and plasticity of macrophages that is in part dictated by the cytokine signals [198], [199], [200]. A number of macrophage phenotypes have been identified (e.g. M1, M2, M4) with the two major ones being classically activated M1 (thought to be derived from Ly6C^high^-monocytes and produces pro-inflammatory cytokines such as IL-6, IL-12 and TNF-α) and the alternatively activated M2 (thought to be derived from Ly6C^low^-monocytes and produces anti-inflammatory cytokines such as IL-10 and TGF-β) that aid in the resolution of inflammatory responses [2], [198], [199], [200]. T-helper (Th)-1 cytokines such as IFN-γ and IL-1β favor M1 differentiation whereas Th2 cytokines such as IL-4 and IL-13 are required for M2 differentiation [198], [199], [200]. The pro-atherogenic roles of IFN-γ and IL-1β are well established (Table 3, Table 4) and recent studies have shown that administration of IL-13 in the LDLr^−/−^ model system drives M2 macrophage polarization and inhibits atherosclerosis progression [121]. However, the role of IL-4 in atherosclerosis is not clear-cut [101], [102].

LDL tends to diffuse passively through EC junctions and its retention is aided by interaction of its apoB moiety with components of the extracellular matrix such as proteoglycans [2]. The accumulated LDL is subject to modification such as aggregation and oxidation. The latter to form oxidized LDL (OxLDL) is in response to exposure to oxidants from the activated endothelium and due to the action of enzymes such as 12/15-lipooxygenase, myeloperoxidases and NADPH oxidases [2], [201]. The effect of some cytokines on the oxidation of LDL by monocytes/macrophages or other cells involved in atherosclerosis along with the expression/activation of enzymes implicated in this process has been investigated though the outcome of these studies have not often been consistent [202], [203], [204], [205], [206]. For example, TNF-α, IL-4 and IL-13 promoted LDL oxidation [202], [203], [204] though contradictory findings were obtained with IFN-γ [205], [206].

Macrophages express pattern recognition receptors (PRRs), such as scavenger receptors (SRs), toll-like receptors (TLRs) and nucleotide-binding oligomerization domain (NOD)-like receptors (NLR), that participate in foam cell formation and/or elicit an inflammatory response against foreign particles or endogenously produced danger signals [2], [14]. The SRs and to a certain extent TLRs play critical roles in foam cell formation. For example, minimally modified LDL (mmLDL) and its components stimulate TLR4-dependent fluid phase uptake of lipoproteins by macrophages [207]. SRs, such as SR-A1 and CD36, recognize modified LDL and uptake these particles to convert into lipid-laden foam cells [2]. The uptake of LDL via the LDLr is under negative feedback regulation through the sterol response element binding protein (SREBP) regulatory pathway [2]. In contrast, the uptake of modified LDL by SRs in not under such negative feedback regulation and therefore causes uncontrolled uptake [2]. Mechanisms other than SRs-mediated endocytosis, such as pinocytosis, phagocytosis and macropinocytosis, also participate in the uptake of lipoproteins, including native and modified LDL [2].

As shown in Table 3, Table 4, several cytokines modulate foam cell formation *in vivo*; for example, IFN-γ and TNF-α promote this whereas IL-1RA and IL-33 are inhibitory. The formation of foam cells involves dysfunction of a homeostatic mechanism that controls the uptake, intracellular metabolism and efflux of cholesterol by macrophages [2]. Several cytokines modulate macrophage foam cell formation by disrupting such a homeostatic mechanism via regulation of the expression and/or activity of key genes implicated in these processes [2]. For example, IFN-γ promotes modified LDL uptake by inducing the expression of the SR that binds phosphatidyl serine and oxidized lipoproteins (SR-PSOX) [208] and our recent studies have shown that the extracellular signal-regulated kinase (ERK)/signal transducer and activator of transcription-1 (STAT1) signaling is required for the macrophage uptake of modified LDL by this cytokine [209]. In addition, the cytokine decreases cholesterol efflux by inhibiting the expression of key proteins involved in this process, such as ATP-binding cassette transporter (ABC)A1 and ApoE, and increasing intracellular levels of chostereyl esters by augmenting the expression/activity of acyl-CoA acyl transferase-1 (ACAT-1), which is involved in the esterification of cholesterol [2], [210], [211]. The TNF superfamily members LIGHT (homologous to lymphotoxin, exhibits inducible expression, and competes with HSV glycoprotein D for herpes virus entry mediator, a receptor expressed by T lymphocytes), TWEAK and TNF-like protein 1A (TL1A) also promote the uptake of modified LDL [2]. For instance, we have shown that TL-1A stimulates macrophage foam cell formation by increasing the uptake of modified LDL and intracellular cholesteryl ester content and decreasing cholesterol efflux [212]. The cytokine induced the expression of several SRs (SR-A, CD36, SR-PSOX) and decreased the expression of ApoE, ABCA1 and ABCG1 [212]. In contrast to these pro-inflammatory cytokines, TGF-β1, IL-10 and IL-33 inhibit macrophage foam cell formation [2]. For example, IL-33 reduces foam cells in the ApoE^−/−^ mice *in vivo* and macrophages *in vitro* by decreasing modified LDL uptake, reducing intracellular content of cholesteryl esters and stimulating cholesterol efflux [148]. These changes were associated with reduced expression of key genes involved in the uptake and intracellular storage of cholesteryl esters, such as SR-A1, CD36, SR-BI, adipocyte differentiation related protein (ADRP) and ACAT-1, and increased expression of those implicated in the intracellular trafficking and efflux of cholesterol [e.g. ApoE, ABCA1, ABCG1, (Niemann-Pick disease, type C (NPC)-1, NPC-2] [148]. TGF-β acts in a similar manner [2], [213], [214], [215]. Additionally, TGF-β and IL-33 attenuate macropinocytosis [216]. On the other hand, IL-10 inhibits foam cell formation by enhancing both modified LDL uptake and cholesterol efflux [117].

## 4 Cytokines and the development of complex lesions

Cholesterol is toxic to cells and mechanisms exist to maintain homeostasis. For example, derivatives of cholesterol, such as oxysterols, activate liver X receptors (LXRs) that then stimulate cholesterol efflux by inducing the expression of ABCA1 and ABCG1 [217]. LXRs also attenuate gene expression associated with inflammation via transrepression [217]. Cytokines, on the other hand, attenuate the actions of LXRs. For instance, IFN-γ inhibits LXR signaling [218] and the expression of LXR-α is decreased during the hepatic acute phase response [219]. Continued inflammation ultimately causes the cholesterol homeostatic mechanisms to become overwhelmed during atherosclerosis. This leads to endoplasmic reticulum (ER) stress in macrophages and, together with other insults, results in cell death by apoptosis and necrosis [2], [14]. Defective clearance of such apoptotic cells (efferocytosis) continues to result in lipid accumulation in the atherosclerotic lesions [2], [14]. Efferocytosis and components involved in the process are also subject to regulation by cytokines [220], [221].

Defective lipid homeostasis is instrumental in the amplification of the inflammatory response and activation of the immune response [14]. Thus, a number of “danger signals” are produced from modification of LDL and lysis of foam cells that activate PRR, such as SRs, TLRs and NLRs, expressed by macrophages [14]. For example, the uptake of cholesterol crystals, which have been observed in early lesions, by macrophages via macropinocytosis activates the NLR family, pyrin domain containing 3 (NLRP3) inflammasome [222]. The process involves lysosomal destabilization and release of reactive oxygen species (ROS) and proteases that then activate NLRP3 inflammasome leading to processing and secretion of IL-1β and IL-18, and subsequent amplification of the inflammatory response [222]. In addition, increases in intracellular cholesterol by other mechanisms (e.g. SRs-mediated uptake) leads to the formation of intracellular cholesterol crystals and activation of NLRP3 inflammasome [223]. Furthermore, mmLDL, which is not recognized by SRs, causes production of pro-inflammatory cytokines via a TLR4-dependent manner [224]. Moreover, OxLDL uptake by CD36 causes expression of cytokines via a mechanism involving TLR2-TLR4 [225].

Antigen presenting cells, such as macrophages and DCs, are essential for the adaptive immune responses as they engulf and process antigens, and present them on the cell surface in association major histocompatibility complexes (MHC) for recognition by T-cells [226], [227]. A range of T-cells exists and many studies have investigated their roles in atherosclerosis [226], [227]. In general, Th1 cells are most abundant in atherosclerotic plaques, secrete cytokines such as IFN-γ, TNF-α and IL-2 and are pro-atherogenic, whereas Tregs, which produce TGF-β and IL-10, are anti-atherogenic [226], [227]. In contrast, the roles of Th2 cells (secrete IL-4, IL-5 and IL-13) and Th17 cells (produce IL-17A/F along with IL-22 and IL-23) are not clear-cut [226], [227]. The actions of some cytokines on atherosclerosis (e.g. IL-10, IL-18, IL-19, IL-33, TGF-β) are mediated, at least in part, by modulating T-cell activation/levels/actions (Table 3, Table 4). Th1 commitment is mainly triggered by IFN-γ and IL-12, IL-6 and IL-13 play an important role in Th2 differentiation, TGF-β and IL-6 are necessary for Th17 differentiation, and generation of Tregs depends on TGF-β [227]. In addition to T-cells, other immune cells, such as DCs, natural killer (NK) cells, NKT cells, subsets of B cells, mast cells and neutrophils play an important role in atherosclerosis and contribute to the inflammatory responses in this disease [226], [227].

SMCs also play an important role in atherosclerosis and more advanced lesions are generally covered with a fibrous cap, composed of SMCs and ECM components produced by them, and lipid-rich necrotic core [2]. Migration of SMCs from the media to the intima along with their proliferation is controlled by various growth factors produced by macrophages, ECs and T-cells [228], [229]. SMCs also express SRs on their cell surface and can uptake modified LDL to form foam cells [228], [229]. Cytokines can modulate the process by regulating the expression of SRs in isolation or synergistically with growth factors [228], [229]. In addition, IL-1β disrupts the cholesterol-mediated LDLr feedback regulation in these cells and produces increased expression of this receptor [230].

## 5 Roles of cytokines in plaque stability and rupture

The continued inflammatory response ultimately leads to the destabilization of atherosclerotic plaques via the action of pro-inflammatory cytokines. Indeed, studies in mouse model systems have shown that IFN-γ, IL-18, GDF-15 and TWEAK destabilize plaques whereas TGF-β causes stabilization (Table 3, Table 4). Cytokines modulate a number of steps in the control of plaque stability and rupture. For instance, some, such as IFN-γ, TNF-α and IL-1β, promote apoptosis of macrophages along with foam cells leading to enlargement of the lipid core [231]. In addition, such cytokines stimulate apoptosis of SMCs leading to thinning of the fibrous cap [228], [232], [233]. Pro-inflammatory cytokines also inhibit the synthesis of plaque stabilizing components of the ECM produced by SMCs [232], [233]. For example, IFN-γ inhibits the synthesis of collagen by SMCs [234]. Further remodeling of the ECM is controlled by a range of proteases, particularly matrix metalloproteinases (MMPs), and their inhibitors (tissue inhibitor of metalloproteinases—TIMPs) produced by macrophages and other vascular cells [235], [236]. The expression and/or activities of MMPs and TIMPs are regulated by cytokines [235], [236]. Vulnerable plaques have very few SMCs and high macrophage content, and are susceptible to rupture leading to thrombosis [2], [232], [233]. Key components involved in thrombosis are also subject to regulation by cytokines. For example, studies in mouse model systems have shown that deficiency of IL-10 is associated with tissue factor (TF) activities together with markers of systemic coagulation and vascular thrombosis [111]. In addition, the interaction of TWEAK with its receptor, fibroblast growth factor-inducible 14 (Fn14), stimulates the expression of plasminogen activator inhibitor (PAI)-1 and TF by SMCs [237]. Furthermore, the production of TF is activated by a number of cytokines such as TNF-α, IL-1, IL-6, IL-8 and IFN-γ [238]. Similarly, the expression of PAI-1 is modulated in inflammatory conditions [239], [240]. Pro-inflammatory cytokines also suppress natural anticoagulant mechanisms, such as the protein C pathway [241].

## 6 Conclusions and therapeutic perspectives

Inflammation plays a pivotal role in all stages of atherosclerosis: endothelial dysfunction; recruitment of immune cells; modification of LDL; foam cell formation; apoptosis of foam cells; plaque rupture; and thrombosis. The inflammatory response in atherosclerosis is regulated by both the innate and adaptive immune system via the action of cytokines. Our understanding of the roles of such cytokines has recently advanced considerably mainly via studies using mouse model systems (summarized in Table 1, Table 2, Table 3, Table 4). Attenuating cytokine-induced inflammation and promoting the actions of anti-inflammatory cytokines represent potential therapeutic avenues. Indeed, statins and athero-protective agonists of nuclear receptors attenuate signaling and gene expression mediated by certain cytokines [217], [242], [243], and this could be responsible, at least, in part for their beneficial effects. In addition, the athero-protective effects of certain agents (e.g. estradiol) on atherosclerosis in mouse model systems have been attributed to TGF-β [177].

A number of anti-inflammatory therapies aimed at manipulating cytokine actions are currently being evaluated [2], [11], [243]. For example, the Cardiovascular Inflammation Reduction Trial (CIRT) has been initiated to evaluate the efficacy of low doses of methotrexate, which has proved beneficial in the treatment of the inflammatory disorders rheumatoid arthritis and psoriasis, for the secondary prevention of myocardial infarction [244]. In addition, a phase II clinical trial of canakinumab, IL-1β neutralizing antibody, reported lowering of inflammatory markers and formed the basis of a larger phase III, secondary prevention Canakinumab Anti-inflammatory Thrombosis Outcomes Study (CANTOS) [243], [245], [246]. A monoclonal antibody (MLN1202) that targets interaction of CCL2 with its receptor has also been evaluated on a smaller scale with reduction in the levels of the acute phase reactant C-reactive protein [247].

Therapeutic approaches need not be restricted to cytokines themselves. For instance, many studies in mouse model systems have demonstrated the protective roles of Tregs and cytokines produced by them (IL-10 and TGF-β) [226], [227]. Many studies are investigating the potential of approaches that augment the levels/action of Tregs or stimulate immune tolerance to antigens associated with atherosclerosis [226], [227]. Manipulating cytokine signaling represents another approach and could include small molecule inhibitors that attenuate the action of pro-inflammatory components or enhance those that are present naturally to dampen inflammation (e.g. suppressors of cytokine signaling—SOCS) [2], [3], [11]. Noncoding RNA, particularly microRNA (miRNA), are emerging as future potential targets in dampening cytokine-mediated inflammation in atherosclerosis [248]. For example, deficiency of mIR155, whose expression is stimulated by miR-342-5p [249], reduces inflammatory responses in mouse model systems [250]. Pro-inflammatory cytokines induces miR-146a/b in ECs, which then acts as a negative feedback loop to control pro-inflammatory signaling in EC activation [251]. Future research will inform on the potential of miRNA therapeutics and refine current approaches aimed at manipulating cytokine actions in atherosclerosis.

## Conflict of interest statement

None.

## Figures and Tables

**Table 1:** The roles of key chemokines in atherosclerosis.

Chemokine	Summary of studies using mouse model systems	Ref.
CCL2	Hematopoietic overexpression in ApoE^−/−^ model accelerates atherosclerosis without affecting lipoprotein profile. Inhibition by transfection of an N-terminal deletion mutant in skeletal muscle of ApoE^−/−^ mice limits progression and destabilization of established plaques and normalizes levels of pro-inflammatory mediators. Deficiency reduces atherosclerosis in ApoE^−/−^ or LDLr^−/−^ models. Local gene silencing using adenoviral vectors promotes plaque stabilization in the ApoE^−/−^.	[18], [19], [20], [21], [22], [23]
CCL3	BMT in the LDLr^−/−^ model system shows that deficiency of CCL3 reduces atherosclerotic burden and decreases accumulation of neutrophils.	[24], [25]
CCL5	Knockdown of Y-box binding protein-1, which controls CCL5 expression, reduces neointima formation following carotid ligation in the ApoE^−/−^ model.	[26]
CXCL5	Inhibition in the ApoE^−/−^ model leads to macrophage foam cell accumulation in atherosclerotic plaques. The chemokine modulates macrophage activation and stimulates cholesterol efflux together with associated changes in gene expression.	[27]
CXCL1	Blocking antibodies increases neointimal formation and inhibits endothelial recovery after carotid injury in ApoE^−/−^ model.	[28]
CXCL10	Deficiency in the ApoE^−/−^ model reduces atherosclerosis by modulating the local balance of effector and regulatory T cells with increased levels of TGF-β and IL-10. Inhibition using neutralizing antibodies in the ApoE^−/−^ produces a stable plaque phenotype.	[29], [30]
CXCL16	Deficiency in the LDLr^−/−^ model exacerbates lesion formation. Overexpression promotes a vulnerable plaque phenotype in the ApoE^−/−^ model.	[31], [32]
CX_3_CL1	Deficiency in the ApoE^−/−^ or LDLr^−/−^ models reduces atherosclerosis in the brachiocephalic artery but not in the aortic root.	[33]
CCL17	Deficiency in the ApoE^−/−^ model reduces atherosclerosis that is dependent on Tregs. Expression of CCL17 by DCs limits expansion of Tregs and enhances atherosclerosis. CCL17 blocking antibody expands Tregs and reduces atherosclerosis.	[34]
CCL19/CCL21	Transplantation of bone marrow from mice lacking both these chemokines in the LDLr^−/−^ model increases inflammatory cellular infiltration but decreases expression of several pro-inflammatory cytokines. Plaque stability is increased but lesion development remains unchanged.	[35]
CXCL12	Administration in the ApoE^−/−^ model promotes a more stable plaque phenotype and enhances the accumulation of smooth muscle progenitor cells without promoting atherosclerosis.	[36]
CXCL4	Elimination from platelets reduces atherosclerosis in C57BL/6 and ApoE^−/−^ mice.	[37]
MIF	Deficiency in the LDLr^−/−^ model reduces atherosclerosis associated with impaired monocyte adhesion to the arterial wall. Blockade in mice with advanced atherosclerosis leads to plaque regression and reduces monocyte and T-cell content in plaques. Blockade in the LDLr^−/−^ model following experimental angioplasty decreases vascular inflammation, cellular proliferation and neointimal thickening. Inhibition in the ApoE^−/−^ model reduces aortic inflammation and, following vascular injury, shifts the cellular composition of neointimal plaques to a stable phenotype with reduced inflammatory cells and increased SMC content.	[38], [39], [40], [41], [42]

**Table 2:** The roles of key chemokine receptors in atherosclerosis.

Receptor	Summary of studies using mouse model systems	Ref.
CCR1	Deficiency in the ApoE^−/−^ model increases plaque area, T-cell content and levels of IFN-γ but doesn’t protect against neointima formation following wire injury.	[43], [44]
CCR2	Deficiency in the ApoE^−/−^ model reduces lesion formation. Transplantation of CCR2 deficient bone marrow in the ApoE^−/−^ model suppresses angiotensin II-mediated acceleration of atherosclerosis and abdominal aortic aneurysm, and in the ApoE3-Leiden model reduces overall atherosclerotic lesion development but has no effect on the progression of established plaques. Pharmacological inhibition reduces macrophage infiltration in the ApoE^−/−^ model expressing human CCR2. Monocyte-targeted RNA interference in the ApoE^−/−^ model reduces recruitment of Ly-6C^high^ monocytes, attenuates inflammation and improves infarct healing.	[22], [45], [46], [47], [48], [49], [50]
CCR5	Deficiency in the ApoE^−/−^ model protects against atherosclerosis and is associated with a more stable plaque phenotype, reduced infiltration of monocytes and decreased Th1 inflammatory response, and increased production of IL-10. An important role in late-stage atherosclerosis was also identified involving modulation of macrophage accumulation in the plaque and reduction in circulating levels of IL-6 and MCP-5. Antagonist attenuates atherosclerosis and reduces myocardial reperfusion injury in mouse models. Transplantation of CCR5 deficient bone marrow in the LDLr^−/−^ model attenuates atherosclerosis with increased IL-10 expression and reduced TNF-α levels.	[43], [51], [52], [53], [54]
CCR6	Deficiency in the LDLr^−/−^ model reduces atherosclerotic burden by affecting monocyte-mediated inflammation. Reduced atherosclerosis also seen in the ApoE^−/−^ model accompanied by decrease in both circulating levels of monocytes and their migration. BMT reveals importance of chemokine expressed by hematopoietic cells.	[55], [56]
CCR7	Expression induced in an atherosclerosis regression model in ApoE^−/−^ mice. Abrogation of function using antibodies against ligands CCL19 and CCL21 preserved lesion size and foam cell content in this model. Deficiency in the LDLr^−/−^ model attenuates atherosclerosis by modulating T-cell entry and exit into lesions. In contrast, deficiency in the ApoE^−/−^ model exacerbates the disease by increasing T-cell accumulation. BMT confirms the importance of CCR7 expressed by hematopoietic cells.	[57], [58], [59]
CXCR2	Transplantation of CXCR2 deficient bone marrow in the LDLr^−/−^ model reduces macrophage content in established plaques.	[60]
CXCR3	Blockade in the LDLr^−/−^ model using the antagonist NBI-74330 inhibits atherosclerosis by reducing activated T-cells and increasing Tregs. Deficiency in the ApoE^−/−^ model reduces early atherosclerotic lesion development in the abdominal aorta associated with upregulation of IL-10, IL-18BP, eNOS and Tregs.	[61], [62]
CXCR4	Functional blockade in the ApoE^−/−^ or the LDLr^−/−^ models promotes atherosclerosis through deranged neutrophil homeostasis. Antagonists reduce neointima formation without impairing endotheliazation following carotid wire injury in the ApoE^−/−^ model. Deficiency of endothelial CXCR4 attenuates reendothelialization and stimulates neointima hyperplasia following vascular injury in ApoE^−/−^ mice.	[63], [64], [65], [66]
CXCR6	Deficiency in the ApoE^−/−^ model decreases plaque formation and reduces T-cell and macrophage content.	[67]
CXCR7	Activation in the ApoE^−/−^ model improves hyperlipidemia by stimulating cholesterol uptake in adipose tissue.	[68]
CX3CR1	Deficiency in the ApoE^−/−^ model decreases atherosclerosis associated with reduced recruitment of macrophages and DCs. Antagonist inhibits atherosclerosis in both ApoE^−/−^ and LDLr^−/−^ models by modulating monocyte trafficking.	[69], [70], [71], [72]

**Table 3:** The roles of interleukins in atherosclerosis.

Interleukin	Summary of studies using mouse model systems	Ref.
IL-1α	BMT in the LDLR^−/−^ model demonstrated that macrophage-derived IL-1α but not IL-1β drives atherosclerosis. BMT in the ApoE^−/−^ model also demonstrated the importance of IL-1α. Fatty acid-induced uncoupling of mitochondrial respiration elicits inflammasome-independent IL-1α production that drives vascular inflammation in atherosclerosis. Active immunization targeting IL-1α decreases both the inflammatory reaction and plaque progression in the ApoE^−/−^ model.	[82], [83], [84]
IL-1β	Deficiency or blocking of the cytokine in the ApoE^−/−^ model decreases atherosclerosis and expression of several pro-inflammatory genes. Deficiency of IL-1 receptor-1 in the ApoE^−/−^ model reduces atherosclerosis but was surprisingly associated with many unexpected features of plaque stability. BMT in this model showed that selective loss of IL-1 in the vessel wall reduces plaque burden.	[85], [86], [87], [88], [89], [90], [91]
IL-1RA	Overexpression in the LDLr^−/−^ model reduces foam-cell lesion size by affecting plasma cholesterol levels. Overexpression in the ApoE^−/−^ model attenuates fatty streak formation. The IL-1/IL-1RA ratio plays a crucial role in controlling vascular inflammation and atherosclerosis. Heterozygous deficiency in ApoE^−/−^ mice enhances early atherosclerotic lesions with increased macrophage content and decreased level of SMCs. Deficiency in the C57BL/6J background promotes neointimal formation after wire injury.	[92], [93], [94], [95], [96], [97]
IL-2	Injection of the cytokine enhances atherosclerosis in the ApoE^−/−^ model whereas injection of anti-IL-2 antibodies reduces this. Cytokine therapy with IL-2/anti-IL-2 monoclonal antibody in this model attenuates atherosclerosis by expanding Tregs and modulating immune-inflammatory components.	[98], [99]
IL-3	Transplantation of bone marrow from mice that are deficient in the common β subunit of the IL-3/GM-CSF receptor in the LDLr^−/−^ model reduces stem cell expansion and monocytosis along with macrophage and collagen content.	[100]
IL-4	Early study showed that deficiency in the ApoE^−/−^ model reduces atherosclerotic lesions. However, another in-depth study involving exogenous delivery and/or genetic deficiency in ApoE^−/−^ or LDLr^−/−^ models showed no involvement in atherosclerotic lesion formation irrespective of the mode of disease induction.	[101], [102]
IL-5	Transplantation of IL-15-deficient bone marrow in the LDLr^−/−^ model reduces levels of IgM that recognizes epitopes in oxidized LDL and accelerates atherosclerosis.	[103]
IL-6	Injection of the cytokine in the ApoE^−/−^ mice increases levels of pro-inflammatory cytokines and lesion size and use of IL-6 lentivirus demonstrated the ability to destabilize plaques. Inhibition of IL-6 trans-signaling using the inhibitor, soluble glycoprotein 130, reduces atherosclerosis by decreasing endothelial cell activation, infiltration of SMCs and recruitment of monocytes. However, deficiency of the cytokine in both ApoE^−/−^ and LDLr^−/−^ models enhanced atherosclerosis.	[104], [105], [106], [107], [108], [109]
IL-10	BMT in the LDLr^−/−^ model showed deficiency of the cytokine accelerates atherosclerosis whereas its overexpression inhibits advanced lesions, decreases cholesterol and phospholipid oxidation products in the aorta along with monocytic activation and produces a shift to Th2 phenotype. Deficiency in the ApoE^−/−^ model increases atherosclerosis associated with increased LDL levels, Th1 response, MMP and tissue factor activities, and markers of systemic coagulation and vascular thrombosis. Deficiency in the ApoE*3-Leiden mice leads to increased neointima surface following cuff-induced stenosis of the femoral artery. A marked inhibition of this along with reduction in plasma cholesterol levels and expression of several pro-inflammatory cytokines was produced by overexpression of IL-10. The cytokine also attenuates the response to wire carotid artery injury in wt mice. Gene therapy using IL-10 encoding viral vectors or plasmids in ApoE^−/−^ or LDLr^−/−^ models reduces atherosclerosis associated with decreased inflammation, oxidative stress, expression of pro-inflammatory markers and macrophage content of plaques.	[110], [111], [112], [113], [114], [115], [116], [117], [118]
IL-12	Blockade of function by vaccination in the LDLr^−/−^ model reduces atherosclerosis with increased SMC and collagen content. Deficiency in the ApoE^−/−^ model reduces lesion. Injection of the cytokine in the ApoE^−/−^ model increases serum levels of anti-oxidized LDL antibodies and accelerates atherosclerosis.	[101], [119], [120]
IL-13	Administration of cytokine in LDLr^−/−^ model promotes favorable plaque morphology by increasing lesional collagen content, decreasing VCAM-dependent monocyte recruitment and inducing M2 macrophage phenotype. Deficiency of the cytokine accelerates atherosclerosis.	[121]
IL-15	Neutralization of the cytokine in the LDLr^−/−^ model using a DNA vaccination strategy reduces plaque size. Blockade of the cytokine increases intimal thickening following carotid artery injury in C57BL/6.	[122], [123]
IL-17	Studies on loss of SOCS3 expression in T-cells of LDLr^−/−^ model demonstrated protective role of the cytokine in atherosclerosis. Transplantation of IL-17 receptor deficient bone marrow in the LDLr^−/−^ model attenuates atherosclerosis whereas deficiency of the cytokine in the ApoE^−/−^ model has no effect on plaque burden but attenuates vascular and systemic inflammation. In contrast, inhibition using neutralizing antibody in the ApoE^−/−^ model prevents atherosclerotic lesion progression by reducing inflammatory burden and cellular infiltration, and improving lesion stability. Similarly, deficiency of the cytokine or its receptor in the ApoE^−/−^ model reduces atherosclerosis and vascular inflammation whereas injection of IL-17 promotes the disease. IL-17 exacerbates ferric chloride-induced arterial thrombosis in rat carotid artery.	[124], [125], [126], [127], [128], [129], [130], [131], [132], [133], [134]
IL-18	Deficiency in the ApoE^−/−^ model reduces atherosclerosis associated with decreased action of IFN-γ, more stable plaque phenotype and shift to Th2 immune response though a pro-atherogenic role was identified in one study. Administration potentiates atherosclerosis associated with elevated levels of IFN-γ and reduced plaque stability. Lack of endogenous IFN-γ ablated the effects of IL-18 on atherosclerosis. *In vivo* electrotransfer of an expression plasmid encoding IL-18 binding protein in the ApoE^−/−^ model attenuates atherosclerosis.	[135], [136], [137], [138], [139], [140]
IL-19	Administration reduces atherosclerosis in the LDLr^−/−^ model by promotiong Th2 polarization, decreasing leukocyte adhesion and suppressing pro-inflammatory gene expression. Also, reduces ligation-mediated neointimal hyperplasia by decreasing activation of SMCs.	[141], [142]
IL-20	Administration in the ApoE^−/−^ model promotes atherosclerosis.	[143]
IL-25	Administration in the ApoE^−/−^ model reduces atherosclerosis via modulation of innate immune responses.	[144]
IL-27	Deficiency of the cytokine or its receptor in the LDLr^−/−^ model along with BMT and *in vitro* cell culture-based approaches showed that IL-27 inhibits atherosclerosis by attenuating macrophage activation, uptake of modified LDL and pro-inflammatory cytokine production. Transplantation of IL-27 receptor deficient bone marrow in the LDL^−/−^ model increases atherosclerotic development by skewing immune responses towards Th17 cells.	[145], [146]
IL-33	Administration in the ApoE^−/−^ model reduces atherosclerosis associated with decreased foam cell content and levels of IFN-γ, and increased levels of IL-4, -5 and -13. Cytokine produced a Th1 to Th2 shift and had higher levels of anti-OxLDL antibodies. Mice treated with a soluble decoy receptor that neutralizes IL-33 developed larger plaques. Action of IL-33 was mediated in a IL-5-dependent manner.	[147], [148]

**Table 4:** The roles of other cytokines in atherosclerosis.

Cytokine	Summary of studies using mouse model systems	Ref.
GDF-15	Transplantation of GDF-15-deficient bone marrow in LDLr^−/−^ model attenuates macrophage chemotaxis and accumulation, and produces a stable plaque phenotype. Deficiency in the ApoE^−/−^ model inhibits atherosclerosis by decreasing apoptotic cells and IL-6-dependent inflammatory response to vascular injury. Transgenic overexpression in ApoE^−/−^ model reveals a protective role.	[149], [150], [151], [152]
G-CSF	Administration in the ApoE^−/−^ model reduces atherosclerosis (2 studies) associated with decreased serum cholesterol, increased circulating monocytes, and increased expression of IL-10 and Tregs. However, one study found increased atherosclerosis by G-CSF.	[153], [154], [155]
GM-CSF	Deficiency in the LDLr^−/−^ model showed that it promoted advanced plaque progression by increasing macrophage apoptosis susceptibility. However, another study found reduced atherosclerosis associated with decreased content of dendritic and T-cells and disruption of elastic fibers adjacent to the lesion. Injection of viral-encoding GM-CSF in the LDLr^−/−^ model increases atherosclerosis associated with oxidative stress, inflammation and adhesion protein expression. Deficiency in the ApoE^−/−^ model increases lesion size associated with accumulation of macrophages and reduction in collagen content. In contrast, administration in the ApoE^−/−^ model exacerbates atherosclerosis.	[114], [156], [157], [158]
IFN-α	Administration accelerates atherosclerosis in the LDLr^−/−^ model.	[159]
IFN-β	Administration promotes atherosclerosis in ApoE^−/−^ and LDLr^−/−^ models. In contrast, the cytokine attenuated angiotensin II-accelerated atherosclerosis and vascular remodeling in ApoE^−/−^ model.	[160], [161]
IFN-γ	Deficiency of receptor in ApoE^−/−^ model reduces atherosclerosis lesion size and lipid accumulation, and increases collagen content. Deficiency of the cytokine attenuates atherosclerosis in ApoE^−/−^ or LDLr^−/−^ mice and BMT in this model reveals the importance of cytokine expressed by the hematopoietic compartment. Administration in the ApoE^−/−^ model increases atherosclerosis associated with elevated levels of T-cells. Postnatal blocking of the function of the cytokine in the ApoE^−/−^ model via overexpression of soluble decoy receptor prevents atherosclerotic plaque formation and stabilizes advanced plaques.	[162], [163], [164], [165], [166], [167], [168]
M-CSF	Deficiency in the ApoE^−/−^ or LDLr^−/−^ models attenuates atherosclerosis.	[169], [170], [171], [172]
TGF-β	Gene therapy in LDLr^−/−^ mice reduces atherosclerosis associated with decreased oxidative stress, inflammation and adhesion protein expression. Inhibition of TGF-β signaling in the ApoE^−/−^ model accelerates atherosclerosis associated with increased inflammation and decreased collagen content. BMT reveals the importance of the cytokine expressed by the hematopoietic compartment. Overexpression in the ApoE^−/−^ mice reduces atherosclerosis by decreasing T-cell and macrophage content and inflammatory cytokines, and increasing collagen levels. The protective effect of estradiol on fatty streak formation in the ApoE^−/−^ model requires TGF-β. Disruption of TGF-β signaling in dendritic and T cells affects atherosclerosis though one study found no effect in relation to T cells.	[173], [174], [175], [176], [177], [178], [179], [180], [181], [182]
TNF-α	Deficiency of the cytokine in the ApoE^−/−^ or APOE*3-Leiden models reduces atherosclerosis associated with decreased foam cells and expression of several pro-inflammatory markers. BMT reveals important role of cytokine expressed by the hematopoietic compartment. Transplantation of bone marrow deficient in p55 TNF receptor in the LDLr^−/−^ model reduces atherosclerosis associated with decreased foam cells and expression of pro-inflammatory markers.	[93], [183], [184], [185], [186], [187], [188], [189], [190], [191]
TNFSF12/TWEAK	Genetic deficiency/inhibition in the ApoE^−/−^ model reduces atherosclerosis associated with diminished pro-inflammatory response and enhanced plaque stability.	[192]
TRAIL	Deficiency in the ApoE^−/−^ model accelerates atherosclerosis, vascular calcification, diabetes and plaque instability. Systemic administration reduces atherosclerosis in this model.	[193], [194], [195], [196]
